# Dynamics of the Composition of *Plasmodium* Species Contained within Asymptomatic Malaria Infections in the Central Region of Ghana

**DOI:** 10.1155/2021/7419548

**Published:** 2021-02-24

**Authors:** Dorcas Bredu, Dickson Donu, Linda Eva Amoah

**Affiliations:** ^1^Immunology Department, Noguchi Memorial Institute for Medical Research, University of Ghana, Accra, Ghana; ^2^West Africa Center for Cell Biology of Infectious Pathogens, University of Ghana, Accra, Ghana

## Abstract

**Background:**

Monitoring changes in the composition of the *Plasmodium* species circulating within the population over a period can inform appropriate treatment recommendations. This study monitored variations in the prevalence of four common human *Plasmodium* species carried by children with asymptomatic malaria infections over a two-year period.

**Methods:**

Two cross-sectional studies were conducted in November 2017 and December 2019. A total of 210 children aged between 4 and 13 years were recruited in 2017, and 164 similarly aged children were recruited in 2019. Approximately 150 *μ*l of finger-pricked blood was used to prepare thick and thin blood smears as well as spot Whatman® #3 filter paper. Genomic DNA was extracted from the dried blood spots and used in PCR to amplify the 18S rRNA gene from four different human *Plasmodium* parasites.

**Results:**

Parasite prevalence by microscopy and the prevalence of *P. falciparum* detected by PCR was relatively similar at the two time points (Pearson chi-square = 0.405, *p*=0.525, and Pearson chi-square = 0.452, *p*=0.501, respectively). However, the prevalence of PCR detectable *P. malariae* increased by 8.5-fold, whilst *P. ovale* increased from 0 to 9% in the children sampled in 2019 relative to the children sampled in 2017. The only parasite species identified by microscopy in this study was *P. falciparum*, and no *P. vivax* was identified by either microscopy or PCR in the study population during the study period.

**Conclusion:**

There is the need to implement molecular diagnostic tools for malaria parasite surveillance in Ghana. This will enable the identification and treatment of all circulating malaria parasites including *P. malariae* and *P. ovale,* whose population is expanding in parts of Ghana including Simiw.

## 1. Introduction

All human malaria parasite species are capable of causing symptomatic as well as asymptomatic infections [1]. Symptomatic malaria is predominantly caused by high-density parasite infections [2], whilst asymptomatic infections are predominantly caused by submicroscopic densities of parasites [3]. Asymptomatic malaria parasite carriage can persist for long periods of time without treatment, during which the parasite can continuously produce gametocytes that fuel then malaria transmission [4]. Asymptomatic infections are prevalent in both low and high malaria-endemic regions and are important reservoirs for sustaining malaria transmission [4].

The gold standard for the diagnosis of symptomatic malaria in endemic areas is microscopy. However, the inability of microscopy to detect low-density infections [5–7] does not make it ideal for the detection of *Plasmodium* parasites contained in asymptomatic infections [8]. A number of studies that have used molecular approaches such as polymerase chain reaction (PCR) to assess human malaria parasite carriage have shown that the prevalence of malaria parasites, especially *P. malariae* and *P. ovale*, is greater than previously reported [9, 10]. Infections containing *P. malariae* and *P. ovale* most often present as low-density asymptomatic infections that require molecular tools for accurate detection [11, 12].

The prevalence of the non-*falciparum Plasmodium* parasites including *P. ovale* and *P. malariae* is less common than *P. falciparum* in sub-Saharan Africa. Although *P. falciparum* is the leading cause of symptomatic malaria in sub-Saharan Africa, including Ghana [13], *P. malariae* and *P. ovale* can cause chronic infections [14] and have contributed significantly to anemia and morbidity in some populations, including individuals living in Gabon [15]. Some studies from the Central, Eastern, and Greater Accra Regions of Ghana have reported asymptomatic *P. falciparum* prevalence to be as high as 31%, 32%, and 44%, respectively [7, 9].

Successful malaria control interventions are expected to reduce the prevalence and density of malaria parasites circulating within a population over a period of time. The effectiveness of malaria control interventions implemented in a country or community can therefore be evaluated by determining changes in the prevalence and density of malaria parasites contained within infected individuals over time. An earlier report from a *P. falciparum-*endemic country, Tanzania, has suggested that a decrease in the prevalence of *P. falciparum* can result in an increase in the prevalence of non-*falciparum* malaria parasites [16]. This suggests that the enhanced control of *P. falciparum* in Ghana over the years could result in an increase in the prevalence of the non-*falciparum* malaria parasites due to a decline in the prevalence of *P. falciparum*. In this study, we sought to determine the changes in the prevalence of four common human malaria parasite species carried by asymptomatic children living in Simiw, a community in the Central Region of Ghana over a two-year period.

## 2. Materials and Methods

### 2.1. Ethics Approval and Consent to Participate

The Institutional Review Board of the Noguchi Memorial Institute for Medical Research granted ethical clearance for this study (IRB Approval #: 009/15-16). Permission to conduct this study was also obtained from the Komenda Edina Eguafo Abrem (KEEA) Municipal Director of Education. The study was explained to the parents and or guardians of the school children as well as the school children through a series of interactive meetings. Parents/guardians who were willing to enroll their children into the study provided written parental consent prior to the child being recruited into the study. Children aged 12 years and above were made to endorse a child assent form.

### 2.2. Study Type, Site, and Sample Collection

**Figure 1:**
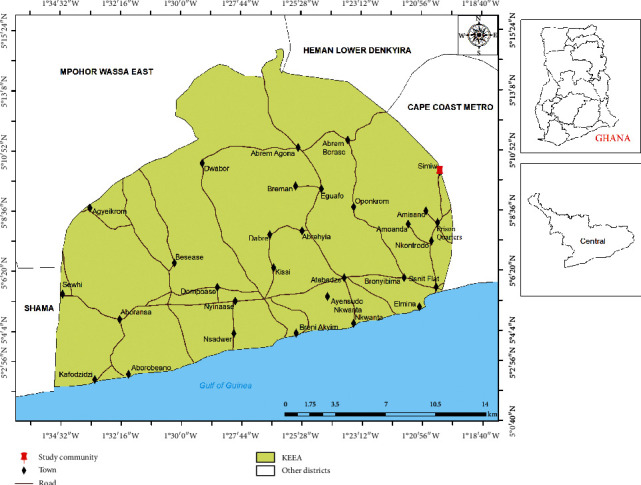
Map of Ghana highlighting Simiw in the KEEA district of the Central Region.

Two cross-sectional studies were conducted over a two-year period. The first was conducted in November 2017, where 210 children aged between 4 and 13 years were enrolled. The second study was conducted in December 2019 where 164 children aged between three and 13 years were recruited. The study was conducted in Simiw, a community in the KEEA District of the Central Region of Ghana (Figure 1). Recruitment of study participants was done from the Simiw Basic School at both time points. All children aged between 3 and 13 years were illegible to be enrolled into the study.

Approximately 150 *μ*l of finger-pricked blood was obtained from each child. The blood was used to prepare a thick and thin blood film as well spot a strip of Whatman® #3 filter paper (GE Healthcare, USA). The blood films were processed for microscopy, whilst the blood spots were air-dried and stored in a Ziplock® bag containing silica gel beads. The samples were then transported to the NMIMR for onward processing and malaria parasite identification.

### 2.3. Malaria Parasite Identification and Quantification by Microscopy

The blood films were processed using the standard WHO protocol [17] and read by two trained malaria microscopists. *Plasmodium* parasite density (PD) was estimated by multiplying the number of parasites identified per 200 WBCs by 40 [5]. A thick smear was considered negative for *Plasmodium* parasites if no parasite was observed in 200 high-powered fields. Discordant slides in terms of presence or absence of *Plasmodium* parasites were re-examined by a third malaria microscopist. Discordant results agreeing with the third reading were considered final.

### 2.4. DNA Extraction

Genomic DNA was extracted from the dried filter paper blood spots (DBS) using the Chelex-saponin extraction method as previously described [18] with very minor modifications. In brief, two 3 mm disks punched from each dried blood spot. The spots were placed in a 1.5 ml microcentrifuge tube and washed with ice cold phosphate buffered saline (pH, 7.2). A 200 *μ*l aliquot of 6% Chelex-100 was then added to each spot and heated at 95°C for 10 minutes. Finally, tube containing the heated mixture was centrifuged at 12,000 × *g* for 1 minute, and 120 *μ*l of the supernatant containing the extracted DNA was transferred into a new sterile labeled 0.5 ml microcentrifuge tube. The DNA was stored at −20°C or used immediately.

### 2.5. Malaria Parasite Identification by PCR

The conserved regions of the *18s rRNA* gene of *P. falciparum*, *P. malariae*, and *P. ovale* were amplified from extracted genomic DNA using a previously described PCR protocol [9]. The *18s rRNA* gene of *P. vivax* was amplified using a similar protocol [19]. The 15 *μ*l primary reaction mixture contained 20–40 ng of DNA, 80 nM of the genus specific primer rPLU5 (forward) and rPLU6 (reverse), 1X PCR buffer, 167 nM dNTP mix, 2.5 mM MgCl_2_, and 1 U of OneTaq DNA polymerase.

Four independent nested (secondary) reactions were performed, with each set up containing a similar composition of reagents as the primary reaction; however, the template used was 0.5 *μ*l of the primary reaction product, and the primer set used was one of the four species-specific primers rFAL1/rFAL2 (133 nM), rMAL1/rMAL2 (333 nM), rOVA1/rOVA2 (333 nM), and rViv1/rViv2 (333 nM) (Supplementary Table S1).

The primary and nested PCR reaction cycling conditions included an initial denaturation at 95°C for 5 minutes followed by 35 cycles of 94°C for 30 seconds, 55°C (for primary) or 58°C (for nested) for 1 minute, 68°C for 1 minute, and a final extension at 68°C for 5 minutes. The nested PCR products were resolved on a 2% agarose gel prestained with ethidium bromide and subsequently visualized on a Vilber gel documentation system (Vilber, France).

### 2.6. Data Analysis

IBM SPSS ver 22 was employed in the descriptive statistics such as frequency and also to identify significant difference between parasite prevalence and PCR-detectable *P. falciparum*, *P. malariae*, and *P. ovale* (Fisher's exact test) in the two sites.

The results were either described as a whole or categorized into young children aged below 10 years and older children aged 10 years and above. The independent samples *T*-test was used to identify differences in the mean age and parasite densities. Differences in the prevalence of males as well as the prevalence of the different *Plasmodium* species detected by PCR were determined using Fisher's exact test. *p* values less than 0.05 were considered statistically significant.

## 3. Results

There were a total of 374 children aged between 3 and 13 years recruited into this study. Although the age range of the children recruited in 2017 and 2019 were similar, the children recruited in 2019 had a higher mean age than those recruited in 2017 (Table 1). There was no significant difference in the prevalence of asymptomatic parasite carriers detected by microscopy (25.7% in 2017 and 28.7% in 2019) or the parasite densities contained in the infections (geometric mean 614 in 2017 and 727 in 2019). The proportion of males in the study population recruited in 2017 and 2019 was also similar and ranged between 49.5% in 2017 and 40.2% in 2019 (Table 1).

### 3.1. Microscopy Estimation of Parasite Prevalence and Species

Overall, the prevalence of asymptomatic malaria infections detected by microscopy in 2017 and 2019 was similar (Fisher's exact test, *p*=0.56) (Table 1). Although the maximum parasite density identified in the samples from 2019 was almost double that of the 2017 samples, the geometric mean parasite densities in the children recruited in 2017 and 2019 samples were similar (Table 1). The only *Plasmodium* species identified by microscopy in this study was *P. falciparum*.

**Figure 2:**
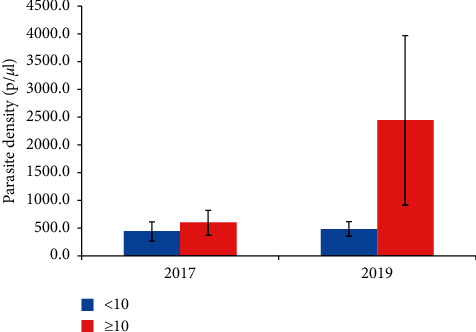
Parasite density estimated by microscopy. The density of parasites estimated from each of the thick smears. <10, children under 10 years (blue bars); ≥10, children aged 10 years and above (red bars). The data are presented as the mean of the parasite density, and the error bars represent the standard error of the mean.

When the children were grouped into young children aged below 10 years (*N* = 150 in 2017 and *N* = 35 in 2019) and older children aged 10 years and above (*N* = 60 in 2017 and *N* = 129 in 2019), the parasite density in the children aged below 10 years in both 2017 and 2019 years was similar. However, a significant increase in parasite density was observed in children aged 10 years and above in 2019 relative to similarly aged children in 2017 (Figure 2 and Table 1).

### 3.2. Polymerase Chain Reaction

Overall, *P. falciparum* species-specific PCR identified 61.4% (129/210) and 57.9 (95/164) of the children recruited in 2017 and 2019, respectively, to have asymptomatic infections (Table 2). No significant difference was observed in the overall number of children or the number of children under the age of 10 years infected with *P. falciparum* in 2017 and in 2019 (chi-squared test, *p*=0.524 and *p*=0.109, respectively). There was a significant increase in the number of children aged 10 years and above that were infected with *P. falciparum* in 2019 relative to similarly aged children 2017 (Table 2).

**Figure 3:**
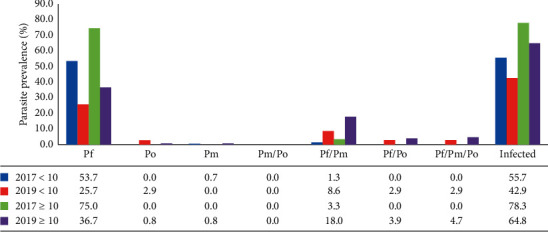
Distribution of the different *Plasmodium* parasites. The prevalence of mono and mixed *Plasmodium* species contained in infections identified in 2017 and 2019. Pf, *P. falciparum*; Po, *P. ovale;* Pm, *P. malariae;* infected, all *Plasmodium* parasites; <10, children less than 10 years; ≥10, children aged 10 years or greater. *Plasmodium vivax* was excluded from the graph because no sample tested positive after the *P. vivax*-specific PCR. The data represent the % prevalence of the overall number of children sampled in each group at each time point.

*Plasmodium malariae*-specific PCR identified 2.4% (5/210) of children from 2017 and 20.7% (34/164) of children from 2019 to harbor asymptomatic infections. In 2017 as well as 2019, a significantly higher number of older children (≥10 years old) tested positive for *P. malariae* relative to the younger children (<10 years) (Table 2). *Plasmodium malariae* monoinfections were identified in 20% (1/5) and 3% (1/34) of the children from 2017 and 2019, respectively (Figure 3). The remaining 80% (4/5) and 97% (33/34) were contained as mixed infections with *P. falciparum* (Figure 3 and Supplementary Table S2).

None of the samples collected in 2017 was identified as positive by the *P. ovale* species-specific PCR; however, 9.1% (15/164) of the children tested in 2019 had *P. ovale*. A majority (87%; 13/15) of the *P. ovale* infections were contained in mixed infections with *P. falciparum*.

The difference in parasite prevalence between children under 10 years old and those 10 years old and above was similar over the study duration, although smaller in 2017 (22.6%) than in 2019 (21.9%) (Figure 3 and Supplementary Table S2). The children aged 10 years and above had a higher prevalence of all the different parasite species observed in the study than the younger children (<10) (Figure 3 and Supplementary Table S2).

## 4. Discussion

The National Malaria Control Program of Ghana has implemented numerous vector control interventions including enhanced distribution of insecticide-treated materials and adulticiding and larviciding of mosquitoes in addition to parasite control interventions including adapting the T3: Test, Treat, Track Initiative. These interventions have resulted in a reduction in the incidence and prevalence of severe manifestations of malaria and uncomplicated malaria but not asymptomatic malaria [20]. The escalation of global efforts to eliminate malaria [21, 22] however requires improved rapid and effective diagnosis of symptomatic as well as asymptomatic infections caused by all human malaria parasites [23, 24].

This study determined the prevalence of children harboring *P. falciparum* in asymptomatic infections to have remained stable over the two-year study period. This supports an earlier report that malaria control in several parts of sub-Saharan Africa, including Ghana, remains a substantial challenge [10]. Behavioral practices of a population, including delayed healthcare seeking behavior [10, 25] and the use of untreated bed nets by some groups of people in Ghana [26], have been suggested to account for a lack of a reduction in the prevalence of malaria despite enhanced nationwide malaria control interventions. Additionally, the continued emergence of drug-resistant parasites and insecticide-resistant mosquitoes [13] as well as the presence of fake and substandard drugs on the market could have added to the persistence of parasitaemic individuals.

The significant increase in the prevalence of *P. malariae* over the two-year period is likely a result of *P. malariae* presenting primarily as asymptomatic infections, which are not treated or symptomatic infections that are treated with alternative antimalarials other than the first-line ACTs. The WHO recommended treatment for *P. malariae* and *P. falciparum* is artemisinin-based combination therapy (ACTs), which when taken appropriately should clear both *P. falciparum* and *P. malariae*. The WHO recommended treatment for *P. ovale* or *P. vivax* is an ACT plus primaquine to clear the hypnozoites [27]. The absence of antimalarials that are effective against the hypnozoites of *P. ovale* on the Ghanaian market [28] is likely to be a major contributor to the increased prevalence of *P. ovale* in Simiw. The hypnozoite stage of *P. ovale* is known to become active after several days to months of dormancy. Infections with *P. malariae* and *P. ovale* most often present as asymptomatic infections [29], which are usually not detected and thus remain untreated [30]. A previous study conducted in the Eastern Region of Ghana also identified a high prevalence of children with asymptomatic infections containing both *P. ovale* and *P. malariae* [9], suggesting a probable increase in non-*falciparum* malaria parasites across Ghana and not just in Simiw. The trend of an increase in parasite carriage by older children relative to younger children has also been identified in studies from other parts of Ghana including the Eastern Region [9] and in other parts of Africa including Equatorial Guinea [31] and Uganda [32]. Younger children are suggested to have a lower incidence of asymptomatic malaria parasite carriage but a higher incidence of symptomatic malaria relative to the older children due to their lower levels of immunity compared to the older children [33].

*Plasmodium malariae* and *P. ovale* parasites were predominantly identified in mixed species infections containing *P. falciparum*. Malaria infections containing *P. falciparum* in addition to a non-*falciparum* parasite is a common occurrence in many tropical regions where *Plasmodium falciparum* is endemic [34] including Ghana [20] and Uganda [35].

## 5. Conclusion

There is the need to implement the use of molecular diagnostic tools for malaria parasite surveillance. This will enable the identification and treatment of all infecting malaria parasites including *P. malariae* and *P. ovale,* whose population is expanding in parts of Ghana including Simiw.

## Figures and Tables

**Table 1:** Demographic characteristics of study participants.

	November, 2017	December, 2019	*T*-test (*p* value)
Overall			
Male (*n*/*N*, %)	104/210 (49.50)	66/164 (40.20)	0.076^*∗*^
Age (yrs) (GM: 95% CI)	7.4 (7.1–7.7)	9.9 (9.4–10.4)	<0.0001
Microscopy (*n*/*N*, %)	54/210 (25.70)	47/164 (28.70)	0.558^*∗*^
PD (*p*/*μ*l) (GM: 95% CI)	613 (446–1165)	727 (480–929)	0.445

<10			
Microscopy (*n*/*N*, %)	14/60 (23.3)	35/129 (27.1)	0.58
PD (*p*/*μ*l) (GM: 95% CI)	588 (339–1043)	1220 (410–4064)	0.076

≥10			
Microscopy (*n*/*N*, %)	40/150 (26.7)	12/35 (34.3)	0.38
PD (*p*/*μ*l) (GM: 95% CI)	694 (286–1824)	608 (348–1042)	0.9

PD, parasite density; yrs, years; GM, geometric mean; 95% CI, 95% confidence interval; *n*, number of samples; *N*, total number of samples; microscopy, microscopy positive samples; *M*, male participants. ^*∗*^*p* value calculated using Fisher's exact test.

**Table 2:** Prevalence of PCR-detectable parasites in the study population.

	2017	2019	*p* value
Overall			
*P. falciparum* (*n/N*, %)	129/210 (61.4)	95/164 (57.9)	0.524
*P. malariae* (*n/N*, %)	5/210 (2.4)	34/164 (20.7)	<0.0001
*P. ovale* (*n/N*, %)	0/210 (0)	15/164 (9.1)	<0.0001

<10			
*P. falciparum* (*n/N*, %)	82/149 (55)	14/35 (40.9)	0.109
*P. malariae* (*n/N*, %)	3/150 (2)	4/35 (11.4)	0.025
*P. ovale* (*n/N*, %)	0/150 (0)	3/35 (8.6)	0.006

≥10			
*P. falciparum* (*n/N*, %)	47/60 (78.3)	81/128 (63.3)	0.039
*P. malariae* (*n/N*, %)	2/60 (3.3)	30/128 (23.4)	<0.0001
*P. ovale* (*n/N*, %)	0/60 (0)	12/128 (9.4)	0.01

<10, children less than 10 years; ≥10, children aged 10 years or greater; *n*, number of samples; *N*, total number of samples.

## Data Availability

All data generated or analyzed during this study are included in this published article.

## Abbreviations

*P. falciparum*: Plasmodium falciparum
*P. malariae*: Plasmodium malariae
*P. ovale*: Plasmodium ovale
DBS: Dried blood spot
PD: Parasite density
ACTs: Artemisinin combination therapy.

## Supplementary Materials

Supplementary Materials Table S1: list of primers and their properties. Table S2: distribution of *Plasmodium* mono and mixed species infections in the study population.
