# Supplementary material for: Dynamics of the Composition of Plasmodium Species Contained within Asymptomatic Malaria Infections in the Central Region of Ghana
Source: J Trop Med. 2021 Feb 24;2021:7419548. doi: 10.1155/2021/7419548 (PMC7936885; doi:10.1155/2021/7419548)
Supplement: Supplementary Materials — Table S1: list of primers and their properties. Table S2: distribution of Plasmodium mono and mixed species infections in the study population. [file 7419548.f1.docx]

**Supplementary files**

Supplementary Table S1. List of primers and their properties

| **Species** | **Primer** | **Primer Sequence (5’-3’)** | **Size (bp)** |
| --- | --- | --- | --- |
| *Plasmodium spp* | rPLU6 | TTA AAA TTG TTG CAG TTA AAA CG | 1050 |
|  | rPLU5 | CCT GTT GTT GCC TTA AAC TTC |  |
| *Plasmodium falciparum* | rFAl1 | TTA AAC TGG TTT GGG AAA ACC AAA TAT ATT | 205 |
|  | rFAL2 | ACA CAA TGA ACT CAA TCA TGA CTA CCC GTC |  |
| *Plasmodium malariae* | rMAL1 | ATA ACA TAG TTG TAC GTT AAG AAT AAC CGC | 144 |
|  | rMAL2 | AAA ATT CCC ATG CAT AAA AAA TTA TAC AAA |  |
| *Plasmodium ovale* | rOva1 | ATC TCT TTT GCT ATT TTT TAG TAT TGG AGA | 787 |
|  | rOva2 | GGA AAA GGA CAC ATT AAT TGT ATC CTA GTG |  |
| *Plasmodium vivax* | rVIV1 | CGCTTCTAGCTTAATCCACATAACTGATAC | 120 |
|  | rVIV2 | ACTTCCAAGCCGAAGCAAAGAAAGTCCTTA |  |

Supplementary Table S2. Distribution of *Plasmodium* mono and mixed species infections in the study population.

|  | Pf | Po | Pm | Pm/Po | Pf/Pm | Pf/Po | Pf/Pm/Po | Infected |
| --- | --- | --- | --- | --- | --- | --- | --- | --- |
| **2017 (210)** | 125 | 0 | 1 | 0 | 4 | 0 | 0 | 130 |
| <10 (150) | 80.0 | 0.0 | 1.0 | 0.0 | 2.0 | 0.0 | 0.0 | 55.7 |
| ≥10 (60) | 45.0 | 0.0 | 0.0 | 0.0 | 2.0 | 0.0 | 0.0 | 78.3 |
| **2019 (164)** | 56 | 8 | 1 | 0 | 26 | 6 | 7 | 104 |
| <10 (36) | 9.0 | 1.0 | 0.0 | 0.0 | 3.0 | 1.0 | 1.0 | 46.9 |
| ≥10 (128) | 47.0 | 1.0 | 1.0 | 0.0 | 23.0 | 5.0 | 6.0 | 76.7 |

The prevalence of mono and mixed *Plasmodium* species in the children sampled in 2017 and 2019. The data represents the exact counts identified in the total population as well as the young (<10 years old) and older children (≥ 10 years old) groups. Pf, *P. falciparum*; Po, *P. ovale;* Pm, *P. malariae;* Infected, all *Plasmodium* parasites; 2017, all the children sampled in 2017; 2019, all the children sampled in 2019; <10, children less than 10 years old; ≥10, children 10 years old or greater*.* *Plasmodium vivax* was excluded from the table because no sample tested positive after the *P. vivax* specific PCR. The
